# Supplementary material for: Preload Dependency of 2D Right Ventricle Speckle Tracking Echocardiography Parameters in Healthy Volunteers: A Prospective Pilot Study
Source: J Clin Med. 2021 Dec 21;11(1):19. doi: 10.3390/jcm11010019 (PMC8745134; doi:10.3390/jcm11010019)
Supplement: Supplementary file 1 [file jcm-11-00019-s001.zip › jcm-1445431-supplementary.pdf]

Supplementary Table **S1– 2D-conventionnel parameters and 2D-STE parameters in the non-responder group.**

**Non-responder group (n=19)**	**Baseline**	**After FC**	**P value**
Right Ventricle Function			
TAPSE (mm)	25 [22–29]	22 [26–31]	0.53
RV- S’ (cm s^{-1})	15 [13–16]	14 [13–16]	0.64
RV FAC (%)	43 [38–49]	42 [38–45]	0.53
IVA (m s^{-2})	3.2 [2.5–3.7]	2.9 [2.4–3.5]	0.45
RV Strain			
RVFWLS (%)	−25.5 [22.7–29.9]	−24.6 [23–28.6]	0.42
RV4CLS (%)	−22.2 [21.2–26.2]	−23.3 [22.2–25.9]	0.52
TAD			
❖ TAD lateral (mm)	24 [21–25]	25 [21–26]	0.11
❖ TAD septal (mm)	14 [15–17]	16 [13–20]	0.75
❖ RV-LSF (%)	24 [22–26]	25 [21–28]	0.37

Continuous variables are expressed as median [interquartile range]

RV-FAC: right ventricule fractional area change. RV-LSF: right ventricle longitudinal shortening fraction. RVOT: Right ventricular outflow tract. RV4CLS: right ventricle four chamber longitudinal strain. RVFWLS: right ventricle free wall longitudinal strain. TAD: tricuspid annular displacement. TAPSE: tricuspid annular plane systolic excursion. VTI: velocity time integral.

Supplementary Table S2– **Reproducibility of 2D-STE parameters.**

Speckle Tracking Parameters	Intra-Class correlation	95% CI	Inter-Class correlation	95%CI
RVFWLS (%)	0.88	0.5–0.97	0.84	0.37–0.96
RV4CLS (%)	0.924	0.7–0.98	0.92	0.68–0.98
TAD lateral point	0.98	0.93–0.99	0.98	0.94–0.99
TAD septal point	0.96	0.85–0.98	0.91	0.67–0.97

RV4CLS: right ventricle four chamber longitudinal strain. RVFWLS: right ventricle free wall longitudinal strain. TAD: tricuspid annular displacement.
